# Effect of Modified* Pulsatilla* Powder on Enterotoxigenic* Escherichia coli* O101-Induced Diarrhea in Mice

**DOI:** 10.1155/2017/3687486

**Published:** 2017-07-17

**Authors:** Jiankang Yu, Yuetian Zhang, Xu Song, Yi Yang, Renyong Jia, Xu Chen, Kai Sun, Li Li, Xinghong Zhao, Qiankun Cui, Qiuting Fu, Yuanfeng Zou, Lixia Li, Zhongqiong Yin

**Affiliations:** Natural Medicine Research Center, College of Veterinary Medicine, Sichuan Agricultural University, Chengdu 611130, China

## Abstract

MPP can be effective in the treatment of* E. coli* O101-induced diarrhea in mice. MPP can improve the weight loss caused by diarrhea, increase spleen and thymus indices, and reduce the diarrhea index. MPP can reduce the number of WBC, regulate the level of cytokines, and regulate the intestinal microbial flora. These data suggest that MPP is a promising candidate for treatment of* E. coli*-induced diarrhea in humans and animals.

## 1. Introduction

Diarrhea is a general disorder characterized by the discharge of semisolid or watery feces three or more times in a day [1]. It involves an increase of fluidity, volume and frequency of bowel sound, wet stool, and abdominal pain, leading to loss of water and electrolytes [2]. In 2009, diarrhea was estimated to have caused 1.1 million deaths in people aged 5 years old and 1.5 million deaths in children under this age [3]. In terms of etiology, diarrhea can be classified into noninfectious and infectious [4]. Noninfectious diarrhea can be caused by toxins, chronic diseases, or antibiotics, while infectious diarrhea occurs worldwide in humans and is caused by vast numbers of bacteria, viruses, and parasites [5].* Escherichia coli *is the common etiological agent involved in bacterial diarrhea in animal and human. According to the different biological characteristics of pathogenesis,* E. coli* is divided into six categories:* Enterotoxigenic E. coli *(ETEC), enteropathogenic* E. coli *(EPEC), enterohemorrhagic* E. coli *(EHEC), enteroaggregative* E. coli *(EAEC), enteroinvasive* E. coli *(EIEC), and enterodispersion* adhesive E. coli *(DAEC) [6].

ETEC is the leading cause of bacterial diarrhea in humans and farming animals through colonizing the small intestine and producing enterotoxins which disrupt intestinal barrier [7]. Antibiotic therapy for infectious diarrhea is controversial, and the etiological agents are often resistant to several broad-spectrum antibiotics [5]. Nowadays, the traditional Chinese medicines have increasingly received attention, due to the abundant resources, few side-effects, long history of safe use in human, and absence of drug resistance. So, development of traditional Chinese medicine preparations is an alternative approach for the prevention and treatment of some infectious intestinal diseases in humans and farm animals.

*Pulsatilla*, a kind of traditional Chinese medicine, derives from the dried root of* Pulsatilla chinensis* (Bunge) Regel. It has been widely used for treatments of intestinal amebiasis, malaria, vaginal trichomoniasis, bacterial infections, and malignant tumor [8]. Earlier studies demonstrated* Pulsatilla* could also be used to treat dysentery [9]. Bai Tou Weng Tang, a Chinese herb decoction, is* Pulsatilla*-based supplemented with* Coptidis rhizoma*,* Cortex fraxini,* and* Cortex phellodendri chinensis* and has been clinically prescribed for hundreds of years to treat toxicosis [10]. Recent study has demonstrated that Bai Tou Weng Tang decoction contains antibacterial ingredients, but the inhibitory and killing effects of Bai Tou Weng Tang decoction on bacteria are not the single way that it plays a role in defensing bacterial diarrhea [11].

The modified* Pulsatilla* powder (MPP) was based on Bai Tou Weng Tang formula and it was prepared using an optimized extraction and preparation process. This study was aimed to evaluate the therapeutic properties of oral administration of the modified* Pulsatilla* powder (MPP) in* E. coli* O101-induced mice diarrhea model. The potency was measured based on the growth performance, diarrhea score, hematology level, serum cytokine levels, immune organ index, intestinal morphology level, and change of intestinal flora for the purpose of developing a new* Pulsatilla* preparation to treat diarrhea.

## 2. Materials and Methods

### 2.1. Drugs Preparation

The MPP (No: 160315) was made in department of pharmacy Sichuan Agricultural University (Chengdu, China). MPP is composed of* Pulsatilla* (Batch number: 150401),* Coptidis rhizoma* (Batch number: 150315),* Cortex fraxini *(Batch number: 150401), and* Cortex phellodendri chinensis* (Batch number: 150405). They were bought from Xinglin Pharmaceutical Chain Co., Ltd. (Chengdu, China). Pulsatilla oral solution (POS) was bought from Qingdao Tianren Bio-pharmaceutical Co., LTD (Qingdao, China). Physiological saline solution (0.9%, w/v) was used as the solvent control group.

### 2.2. Bacterial Strains and Growth Conditions

*E. coli* O101 freeze-drying powder (CVCC3749) was bought from China Veterinary Microbial Culture Collection (Beijing, China). The powder was grown in Tryptone Soy Broth (TSB; Hangzhou Microbial Reacent Co. Ltd., China). After overnight incubation at 37°C with shaking at 180 rmp/min, bacteria were diluted with fresh TSB followed by another overnight incubation. The bacterial cells were harvested by centrifugation at 5000*g* for 10 min at 4°C, and the pellet was washed twice and resuspended in sterile physiological saline (0.9%, w/v). The concentration of bacterial cell suspension was adjusted to different McFarland standard (bioMerieux, France) for a final density of approximately 5 × 10^8^ CFU/mL, 3 × 10^8^ CFU/mL, 2 × 10^8^ CFU/mL, and 1 × 10^8^ CFU/mL, respectively.

### 2.3. Animals

Young adult males (average weight 22 ± 2 g) and females (average weight 22 ± 2 g) SPF mice were bought from Chengdu Dossy Experimental Animals Co, Ltd. [License No. SCXK (Sichuan) 2015-030]. The mice were kept in well ventilated sterile polypropylene cages in the animal houses of Sichuan Agriculture University (Chengdu, China) and provided with sufficient sterilized water and complete formula feed and housed in a rodent facility at 25 ± 3°C with a 12 h light-dark cycle for acclimatization. All procedures involving animals and their care used were approved by the Ethics Committee of Sichuan Agricultural University. Experiments were started after the mice acclimating for a week.

### 2.4. Ethics Statement

All procedures involving animals and their care in this study were approved by the Ethics Committee of Sichuan Agricultural University according to The Regulation of Experimental Animal Management (State Scientific and Technological Commission of the People's Republic of China, number 2, 1988) and The Interim Measures of Sichuan Province Experimental Animal Management (Science and Technology Bureau of Sichuan, China, number 25, 2013).

### 2.5. Determination of* E. coli* O101 Dose for Challenge

50 mice were randomly divided into five groups, each group containing an equal number of 5 females and 5 males. According to the previous reports, animals were challenged with bacteria through intraperitoneal injection [12]. After infection, the clinical symptoms were identified in 72 h. Finally, we obtained the route and dose for bacteria challenging: each mouse was intraperitoneally injected with 0.25 mL of 2 × 10^8^ CFU/mL bacteria, which was showed in Table 1.

### 2.6. Experimental Design

72 mice were randomly divided into six groups including the normal group, negative control group, positive control group (pulsatilla oral solution), and MPP-treated groups, each group containing an equal number of 6 females and 6 males. Each mouse was intraperitoneally injected with 0.25 mL/10 g of 2 × 10^8^ CFU/mL of* Escherichia coli* except the normal group. Three hours later, MPP-treated groups were administered with 0.2 mL/10 g drug at doses of 150, 100, and 50 mg/mL, respectively, and the mice in the positive control group were fed with* Pulsatilla* oral solution (0.2 mL/10 g) according to the instructions. At the same time, the mice in the other two groups were given equal volume of physiological saline solution (0.9%, w/v). After 5 days of continuous treatment (twice a day), all animals were euthanized under ether anesthesia. All animals in this study were subjected to a full necropsy.

#### 2.6.1. Body Weight

During the test, animal body weight was measured at regular intervals each day (7:00 am) and showed as average for each group throughout the experiment.

#### 2.6.2. Fecal Examination

All animals were treated every morning after the treatment, and then they were placed in individual cages, the floor of which was covered with filter paper that was replaced hourly. The numbers and morphology of the stools were recorded for 6 h. The total amount of feces for 6 h was recorded. The severity of diarrhea was defined using three indices: loose stool incidence rate (LSIR), average loose stool grade (ALSG), and diarrhea index (DI) [13]. LSIR is the ratio of number of loose stools to the total stools within an animal. Loose stool grade (LSG) describes the degree of loose stools, based on the diameter of the stool on the filter papers. LSG was classified into four grades according the diameter of loose stools: Grade 1 (<1 cm), Grade 2 (1~<2 cm), Grade 3 (2~3 cm), and Grade 4 (>3 cm). ALSG is the ratio of the sum of LSG of each loose stool to the total number of loose stools within an animal. DI is the result of LSR multiplying ALSG [14, 15].

#### 2.6.3. White Blood Cell Count

On the fifth day of the experiment, after observing the stool condition, mice were then starved for food (but not for water) for 6 h before being executed. Collected blood samples were collected into a EDTA tube for WBC counts.

#### 2.6.4. Serum TNF-*α* and IL-6 Assay

The sera of 10 blood samples from each group were collected, and the concentrations of TNF-*α* and IL-6 were assayed by using a Mouse ELISA kit according to manufacturer's instructions (Shanghai Reagent Chemical Biological Technology Co. Ltd., China).

#### 2.6.5. Relative Weight of Spleen and Thymus Assay

Animals were executed on the fifth day; the spleen and thymus of 10 mice in each group were dissected and weighed. The relative weight of the spleen and thymus was calculated using the following formula:

  Related weight = organ weight (mg)/body weight (g) [16].

#### 2.6.6. Determination of Cecum Microflora

At the end of treatment, ten mice from each group were randomly sampled and sacrificed (the operation is carried out first after dissection). After sterilizing the body surface with 70% alcohol, the mice were dissected under sterile conditions. All cecum contents were collected into sterile plastic tubes. The cecum contents were collected into sterile plastic tubes. The intestinal contents were stored at −20°C until analysis.

The total number of bacteria of samples in the appropriate dilutions was counted by plate count method. The cecum contents were dissolved in the corresponding volume of sterile physiological saline, and the feces were fully dissolved on a vortex shaker. The cecum contents were diluted by 10-fold gradient and the plates were counted at the respective dilutions, and three dilutions were made for each dilution.* Lactobacilli* spp.,* Bifidobacterium *spp.,* Enterococcus* spp.,* Enterobacterium *spp., and* E. coli* were identified and counted using MRS Agar (Qingdao Rishui Bio-Technology Co. Ltd., China), TPY Agar (Qingdao Hope Bio-Technology Co. Ltd., China), Kanamycin Aesculin Azide Agar (Qingdao Hope Bio-Technology Co. Ltd., China**)**, MacConkey Agar (MAC) (Hangzhou Microbial Reacent Co. Ltd., China), and Violet Red Bile Dextrose Agar (VRBDA) (Qingdao Hope Bio-Technology Co. Ltd., China), respectively [17], and incubation conditions were in accordance with that described by Giannenas et al. (2012). Anaerobic incubation was achieved under anaerobic atmosphere (80% N_2_, 15% CO_2_, and 5% H_2_) without agitation. Viable counts per gram of feces were calculated and expressed as log CFU/mL.

### 2.7. Statistical Analysis

Statistical analysis of data was conducted by analysis of variances (ANOVA) of SPSS PASW Statistics v18.0, and Duncan's Test was used to compare the means when the overall *P* value of the experiment was below the value of significance (*P* < 0.05). Mean values and the standard errors were calculated and presented in chart as coordinate pairs with error bars.

## 3. Result

### 3.1. Body Weight

The changes of body weight in female and male were displayed in Figure 2. The results showed that the body weight of the normal group continued to increase during the experiment. The weight of the* E. coli*-infected group decreased rapidly on the first day after challenge, and then the weight of all mice showed an upward trend. Compared with other groups, the mice in the negative control group had a slower weight gain (*P* < 0.05). The body weight of mice in POS-treated group and MPP-treated groups continued to increase, but lower than the normal group (*P* < 0.05).

On day 2, the middle dose of MPP-treated group had a rapid increase when compared to the other treatment groups in female and male mice (*P* < 0.05). After 5 days, the weight of mice in all groups was lower than the normal group and higher than negative control group. Compared to the treatment groups, the weight gain of female mice in high dose of MPP-treated group was higher than that in other groups, and the weight gain of male mice in middle-dose of MPP-treated groups was significantly higher than that of other groups (*P* < 0.05).

### 3.2. Fecal Examination

The diarrhea index of all groups was shown in Table 2. On day 1, Compared with normal control group, the diarrhea index (DI) in* E. coli*-infected group was significantly increased on day 1 (*P* < 0.05). When compared with negative control group, the DI values in all treatment groups were significantly lower in both female and male mice (*P* < 0.05). When compared with other groups, high dose of MPP-treated group and POS group had lower DI values in female mice, and the lowest DI values were found in high dose of MPP-treated group in male mice (*P* < 0.05). The results showed that both MPP and POS had antidiarrhea effect.

On day 2, the DI values of all treated groups in both female and male mice continued to decrease. The DI value of female mice in high dose of MPP-treated group decreased to 0, and the DI values of male mice in high and middle doses of MPP-treated groups were 0.

On day 3, the DI values of all groups were 0 in the female mice, except for negative control group and POS-treatment group. However, DI values were only found in negative control group in male mice. In the last two days, DI values were not present in all groups of mice (*P* < 0.05).

### 3.3. White Blood Cell Count

The changes of White blood cell count (WBC) were shown in Figure 3. Compared with normal control group, a significant increase in WBC was observed in female and male mice after infection with* E. coli*. Compared to negative control group, the WBC count of treatment groups was significantly decreased in female and male mice (*P* < 0.05). MPP-treated groups had lower WBC number when compared with POS-treated group (*P* < 0.05). In MPP-treated groups, the WBC count decreased with the dose increase in female and male mice, gradually approaching the normal value (*P* < 0.05).

### 3.4. Serum TNF-*α* and IL-6 Assay

The changes in TNF-*α* and IL-6 concentrations were illustrated in Figure 4. In female and male mice, the levels of TNF-*α* and IL-6 in negative control group were significantly higher than those in the normal control group, and in the treated groups, TNF-*α* and IL-6 concentrations were significantly lower than those in negative control group (*P* < 0.05). The concentration of IL-6 in middle dose of MPP-treated group, the high dose of MPP-treated group, and POS group were significantly lower than those in low dose of MPP-treated group, which indicated that MPP high dose group is better than low dose group (*P* < 0.05) (Figure 4). There was no difference in the concentrations of TNF-*α* in all treatment groups (*P* > 0.05).

### 3.5. Relative Weight of Spleen and Thymus Assay

Figure 5 showed the spleen and thymus indices of the mice in each group. A markedly decrease of spleen and thymus indices was observed in female and male mice in negative control group (*P* < 0.05). After treatment with MPP and POS, the thymus and spleen indices were significantly increased (*P* < 0.05). The relative weights of thymus and spleen in the MPP medium-dose and the POS group were higher than those in the other treated groups (*P* < 0.05).

### 3.6. Determination of Cecum Microflora

The results of bacterial counts in cecum are shown in Table 3.* Enterococcus *spp.,* Enterobacteriaceae *spp., and* E. coli* in negative control group was significantly higher than those in the other groups, but the numbers of* Bifidobacterium *spp. and* lactobacilli *spp. were significantly lower than that in the other groups (*P* < 0.05). The number of* Enterococci *spp.,* Enterobacteriaceae *spp., and* E. coli* in middle dose of MPP-treated group and POS-treated group was significantly lower than that in the other treated groups, while the* lactobacilli *spp. and the* Bifidobacterium *spp. were higher than those in the other treated groups (*P* < 0.05). The negative control group had the lowest *B*/*E* values in both female and male mice (*P* < 0.05). For both female and male mice, the middle dose of MPP-treated group has the highest *B*/*E* value among all the groups (*P* < 0.05).

## 4. Discussion

**Figure 1:**
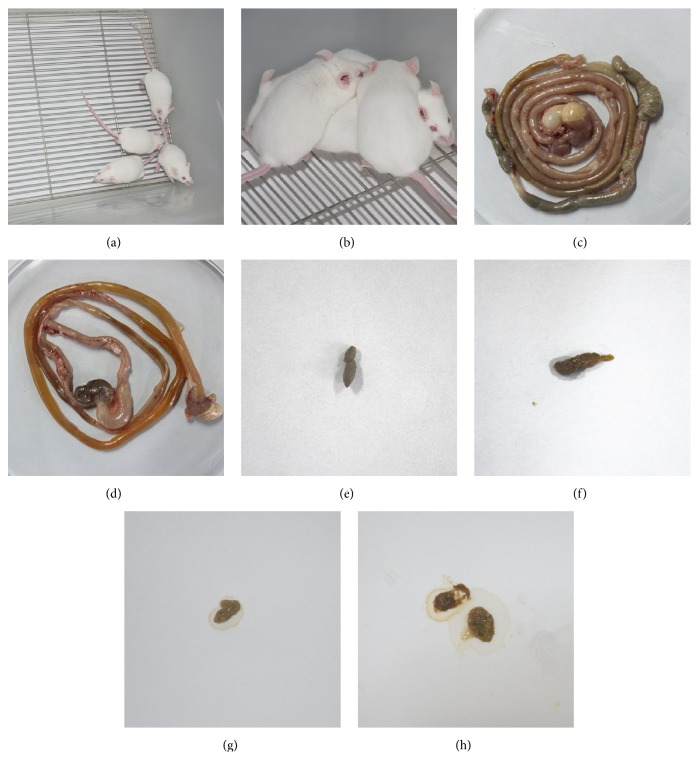
Clinical symptoms of diarrhea mice induced by* E. coli* O101. (a), (c), and (e) showed appearance, feces, and small intestine of normal mice. (b), (d), (f), (g), and (h) showed appearance, feces, and small intestine of diarrhea mice on after infecting by* E. coli* O101.

**Figure 2:**
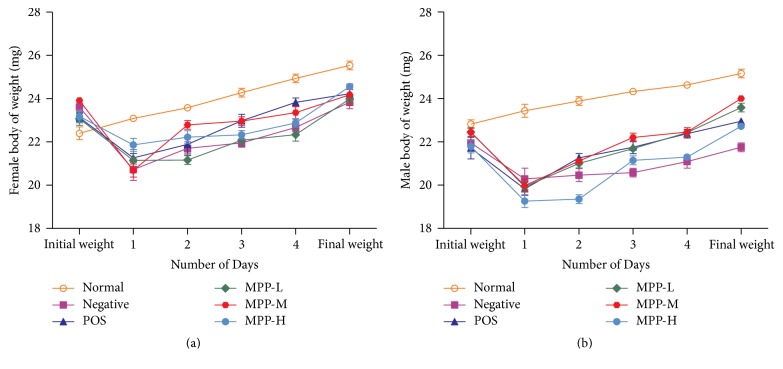
The changes in the body weight of male and female mice in each group during the test. Panel (a) average body weights for female mice during the test. Panel (b) average body weights for male mice during the test.

It is noticed that mice infected with* E. coli *O101 exhibited symptoms 3 h after challenge; clinical symptoms of mice induced by* E. coli* O101 were shown Figure 1, including clinical manifestations of yellow stool around anus, ruffled fur, lethargy, ataxia, tremor, and convulsions before death. Necropsy revealed that the duodenum and jejunal mucosa showed obvious adhesion, congestion, swelling, bleeding, and yellow loose stools in the intestine. A similar performance appears in other studies [18, 19]. These phenomena indicated successful establishment of diarrhea model. This method has the advantages of simple operation and short cycle, so the model could be recommended for screening antidiarrhea drugs.

Modern researches have revealed that the* E. coli* O101 pathogenesis is due to a variety of virulence factors caused by different pathological processes. Endotoxin is a virulence factor, especially in the process of sepsis. It is divided into two categories: heat-labile enterotoxin (LT) and heat-resistant enterotoxin (ST). LT can promote intestinal mucosal cell secretion, resulting in diarrhea and dehydration; ST can increase cGMP production, causing secretory diarrhea [20].* E. coli* can cause dehydration and electrolyte imbalance, significant diarrhea, and decreased weight gain in mice [21]. This study obtained the same results that* E. coli* infection could decrease the weight gain of mice. Treatment with MPP could increase the body weight of infected mice, suggesting MPP were capable of inhibiting weight loss caused by* E. coli* infection.

In previous studies, diarrhea rate can only reflect the proportion of diarrhea in animal groups, but cannot reflect the extent of diarrhea of individual animals. Some studies used “loose stool rate” and “loose stool level” to measure the degree of diarrhea, but these two indicators can measure only one aspect of diarrhea separately. Meanwhile, when the two indicators show opposite results, it is difficult to make an accurate judgment [14, 22]. Studies have shown that the application of diarrhea index in the diarrhea model to reflect the degree of loose stools not only can consider the changes in the amount of loose stools, but also can consider the quality of loose stool, which is more comprehensive and objective than the single use of loose tool rate and loose tool level and can produce more comparable results [13, 14]. The diarrhea index was normal in mice, and statistical treatment could be carried out according to the parameters [15]. Thus, in the present study, diarrhea index was used for evaluation of the degree of diarrhea in mice. The results of our research indicated that MPP significantly reduced the diarrhea index (Table 2), and the effect of high dose of MPP-treated was significantly better than POS.

**Figure 3:**
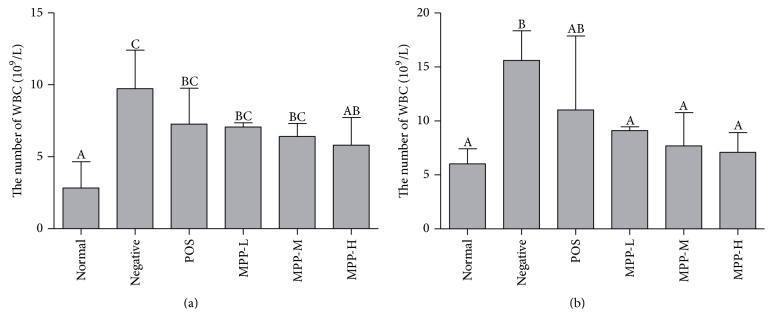
On the changes of WBC counts of MPP. Panel (a) the number of WBC in female mice. Panel (b) the number of WBC in male mice. The data are expressed as the mean ± SD Significant differences were considered at *P* < 0.05. WBC: white blood cell; ^A,B,C^bars in the same cytokine without the same superscripts differ significantly (*P* < 0.05).

WBC count is widely used as inflammatory marker, and the changes of its value is an important reference indicator for the diagnosis and identification of acute infectious diarrhea [23]. Studies have shown that* E. coli* infection significantly increased the total white blood cells in rats [24], which may be due to* E. coli* infection increasing total leukocytes count [25]. In the present study, we also found that intraperitoneal injection of* E. coli* could increase the number of WBC, indicating that the mice suffered acute infectious diarrhea. The results showed that MPP had regulation of abnormal WBC, which is one of the antidiarrhea mechanisms of MPP (Figure 3).

*E. coli* form diarrhea is one kind of inflammatory bowel disease, and IL plays an important role in it. IL is one kind of cytokine receptors with inflammatory mediated activity, which could be divided into many kinds. It is produced mainly by mononuclear macrophages and some activated T cells [26]. Because of its strong inflammatory activity, it could be used directly on vascular endothelial cell to increase its permeability, so as to go through intestinal wall largely to cause a series of clinical symptoms. Meanwhile, it could activate various kinds of inflammatory cells to play a role in decreasing the systemic vascular resistance and producing acute phase protein [27]. Tumor necrosis factor-*α* (TNF-*α*), also known as cachectin, is a cell response factor secreted by monocyte-macrophage and NK cells [28]. Normally, the TNF-*α* activity of body is very low [29]. However, in some pathological conditions, such as the injury of body tissues, especially in acute infectious disease, TNF-*α* is released into local tissue and body fluids are sustained in large quantities, leading to imbalance in other cytokines and producing damage to the body [30]. As the most important proinflammatory factor, the improvement of TNF-*α* will also promote or inhibit the expression and activity of other cytokines. In the pathogenesis of inflammatory bowel disease, the secretion of the proinflammatory cytokine plays an important role, especially TNF-*α* [31]. In all, when you get inflammatory diarrhea, the level of cytokine receptors changed. The anti-inflammatory cytokine receptors and the inflammatory ones influenced each other and join in the producing and development to inflammatory bowel disease. TNF-*α* is the most important proinflammatory factor in the body; its increase will promote or inhibit the expression levels and activity of other cytokines [32]. Changes of IL-6 and TNF-*α* level may be closely associated with the attack, development and even recovery of diarrhea, and the moderate rise of TNF-*α* and IL-6 is conductive to the control over the body inflammatory reaction as well as the prevention from further development of gastroenteritis [33]. However, their excessive increase will lead to the immune injury of the body, worsen the condition, and result in the occurrence of severe illnesses [34].

**Figure 4:**
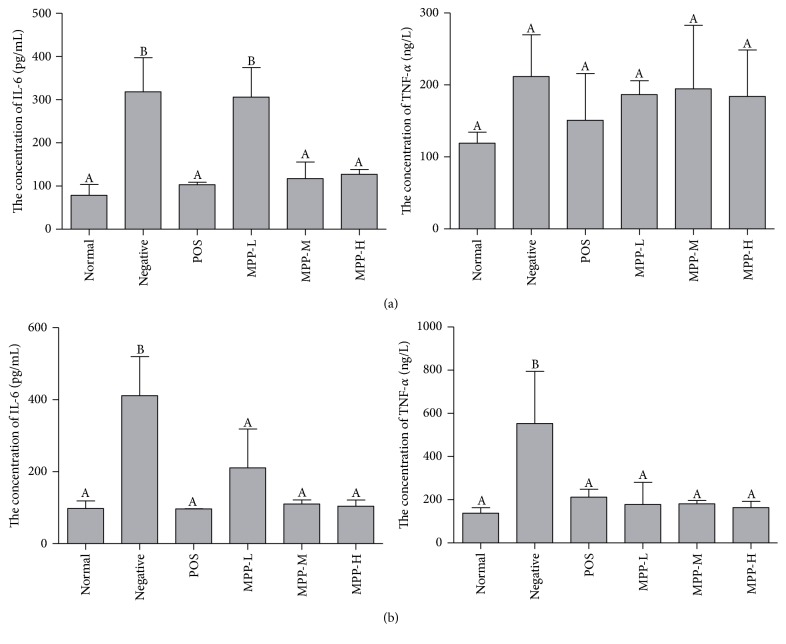
The serum IL-6 and TNF-*α* concentration. Panel (a) the concentration of IL-6 and TNF-*α* in female mice. Panel (b) the concentration of IL-6 and TNF-*α* in male mice. The data are expressed as the mean ± SD Significant differences were considered at *P* < 0.05. ^A,B^Bars in the same cytokine without the same superscripts differ significantly (*P* < 0.05).

Studies have shown that the levels of TNF-*α* and IL-6 in children increased significantly in the acute stage of infectious diarrhea and were positively correlated with the severity of the disease [35]. In this study,* E. coli* infection increased the concentration of IL-6 and TNF-*α*, suggesting that* E. coli *could induce inflammation in mice through IL-6 and TNF-*α* production. MPP-treatment could recover the concentration of IL-6 and TNF-*α* to normal level, which was one of the possible ways for MPP treating diarrhea (Figure 4). The results showed that both MPP and POS could control the inflammation and improve the immune function of mice by downregulating the levels of TNF-*α* and IL-6, so as to effectively treat diarrhea.

**Figure 5:**
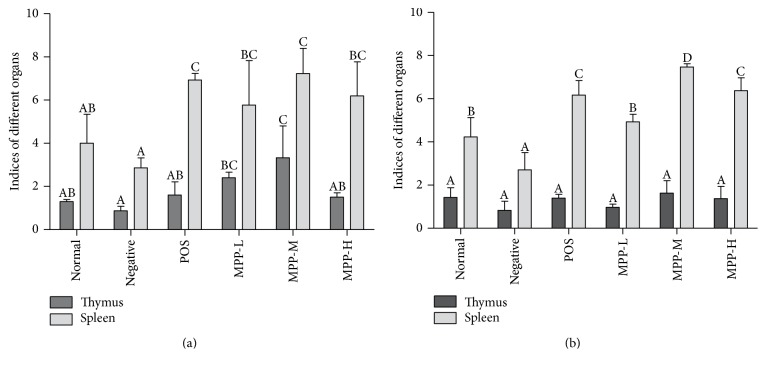
Effect of MPP on indices of thymus and spleen. Panel (a) the indices of thymus and spleen in female mice. Panel (b) the indices of thymus and spleen in male mice. The data are expressed as the mean ± SD Significant differences were considered at *P* < 0.05. ^A,B,C,D^Bars in the same cytokine without the same superscripts different significantly (*P* < 0.05).

The spleen and thymus are the main immune organs of the body. The increase in weight often means that the proliferation of lymphocytes in the body directly reflects the state of immune response [36]. Thymus is closely linked with development, differentiation, and maturation of T lymphocyte [37]. Thymus not only serves as the location of T lymphocytes formation, but also secretes hormones, including thymopoietin and thymosin [38]. Spleen is the organ that can produce lymphocytes, purify blood, and store white cells [39]. The results showed that spleen and thymus index of mice infected with* E. coli* O101 were significantly decreased, indicating that* E. coli* O101 can affect the proliferation of immune cells and reduce the body's ability to immune response [40]. Studies have shown that many herbs have a protective effect on the spleen and thymus. Lentinan, rhizoma atractylodis polysaccharides, and* Cordyceps militaris* polysaccharides could also improve the thymus and spleen index and enhance the immune system of animals [41]. After MPP-treatment, the indices of spleen and thymus were significantly increased, suggesting that MPP could recover the immune function (Figure 5). The results showed that MPP could enhance the anti-infective ability by promoting thymus and spleen growth and promote the recovery of spleen in mice infected with* E. coli *(Figure 5).

An enormous amount of microflora that is relatively stable exists in animals' digestive tract [42]. The diversity of the flora can guarantee the balance of the intestinal microbial community and enhance its adaptability to changeable environment [43]. The microflora can maintain a relatively stable environment of the gastrointestinal tract and facilitate the digestive absorption of nutrient substance [44]. As a living organism, probiotics can sustain gastric acid and intestinal digestive juice and enter the intestinal tract of human body [45]. By virtue of its own growth and proliferation and various kinds of metabolism, it facilitates the normalization of bacterial flora inside intestines and restrains the generation of corruptive substance and the growth of detrimental bacteria. Meanwhile, probiotics can occupy the planting locus of the digestive tract of the host animals, which can reduce the chances of being infected by other microorganisms (pathogeny) [46]. The detrimental bacteria mainly include* Escherichia coli*,* Enteric bacilli*, and* Enterococcus*. As the conditioned pathogen of intestinal tract,* Escherichia coli* weakens the immunity of the organism under the imbalanced flora and triggers intestinal problems [47].* Enteric bacilli *and* Enterococcus* are the conditioned pathogens inside the intestinal tracts [48]. Although the majority of them are normal flora of the intestinal tracts, when the host's immunity is weakened or when the bacteria relocates to the external parts, they can become conditioned pathogen, which will trigger diseases; the minority of them are pathogenic bacteria. As the conditioned pathogen of intestinal tracts,* Escherichia coli *weakens the immunity of the organism under the imbalanced flora and triggers pertinent intestinal problems.* Enteric bacilli* and* Enterococcus* are the conditioned pathogens inside the intestinal tracts, and the majority of them are normal flora of the intestinal tracts [49]. However, when the host's immunity is weakened or when the bacteria relocate to the external parts, they can become conditioned pathogens, which will trigger diseases; the minority of them are pathogenic bacteria [50]. Many studies have shown that traditional Chinese medicine could balance microflora disorder induced by diarrhea through shedding of pathogens and increasing viable counts of total* Lactobacilli* and* Bifidobacterium* [17]. Dietary treatment using L. reuteri HY25101 could reduce diarrheal problem and mortality rate caused by* porcine epidemic diarrhea virus (*PEDV) in suckling pigs [51].* Lactobacillus* plantarum strain Hokkaido isolated from a Japanese kimchi can increase the number of flora in the feces, thereby reducing the incidence of calf diarrhea [52]. Traditionally, the cecal microflora has been analyzed using culture-dependent methods. In our study,* E. coli*,* Enterobacteriaceae *spp. and* Enterococcus *spp. proliferated rapidly in the cecal contents of mice, while the number of viable cells of* Lactobacillus* and* Bifidobacterium* was significantly reduced after infection with* E. coli *O101 (Table 3).* Bifidobacterium *spp. and* Lactobacillus *spp. have generally been recognized as safe status, gaining popularity for application in dairy products and playing probiotic roles in human and animal's gastrointestinal tract [53]. The increased abundance of* Lactobacillus *spp. and* Bifidobacterium *spp. may be associated with improving immunity through activation of TLRs [54]. Furthermore,* Bifidobacterium* spp. species fail to activate the production of key mediators in the inflammatory cascade, including IL-1, IL-6, and TNF-*α*, whereas* E. coli *markedly enhances the synthesis of these products presumably because of endotoxin release [55]. Our results revealed that both MPP and POS increased the number of* Lactobacillus *spp. and* Bifidobacterium *spp. in the mice (Table 3).* Enterobacteriaceae *spp.,* Enterococcus *spp., and* Streptococcus *spp. are recognized as normal parts of the digestive tract flora, which are considered as major opportunistic pathogens that could cause many diseases.* Bifidobacterium *spp. can inhibit the growth of* Enterobacteriaceae* spp.,* Streptococcus *spp. [56], and* Enterococcus* spp. [57]. This study displayed that the number of* Enterobacteriaceae* spp.,* Enterococcus* spp., and* Streptococcus* spp. was decreased by MPP and POS in the mice (Table 3). These results indicated that the treatment effects of MPP and POS may be due to increasing the number of probiotics to inhibit the proliferation of harmful bacteria. We could thus infer that MPP were beneficial in the regulation of intestinal microflora and serve as therapeutic tool for diarrhea in mice.

## Figures and Tables

**Table 1:** Different infection does of *E. coli* O101.

Groups	Animal number	Poison attack way	Dose	Diarrhea rate (%)	Death rate (%)
Group 1 (5 × 10^8^ CFU/mL)	10	Intraperitoneal injection	0.25 mL/10 g	100	100
Group 2 (3 × 10^8^ CFU/mL)	10	Intraperitoneal injection	0.25 mL/10 g	100	80
Group 3 (2 × 10^8^ CFU/mL)	10	Intraperitoneal injection	0.25 mL/10 g	90	10
Group 4 (1 × 10^8^ CFU/mL)	10	Intraperitoneal injection	0.25 mL/10 g	50	0
Group 5 (physiological saline)	10	Intraperitoneal injection	0.25 mL/10 g	0	0

**Table 2:** Effect of MPP on diarrhea index in female and male mice. The values are presented as means ± standard deviation (8 mice/group). ^a–c^Data within a row without the same superscripts are different significantly (*P* < 0.05).

Treatment of days	Normal	Negative	POS	MPP-L	MPP-M	MPP-H
Female						
1 day	0.00 ± 0.00^a^	2.07 ± 0.78^c^	1.12 ± 0.19^b^	1.37 ± 0.20^bc^	1.33 ± 0.58^bc^	1.16 ± 0.29^b^
2 days	0.00 ± 0.00^a^	0.23 ± 0.03^b^	0.09 ± 0.16^a^	0.18 ± 0.17^a^	0.08 ± 0.07^a^	0.00 ± 0.00^a^
3 days	0.00 ± 0.00^a^	0.12 ± 0.02^b^	0.05 ± 0.10^a^	0.00 ± 0.00^a^	0.00 ± 0.00^a^	0.00 ± 0.00^a^
4 days	0.00 ± 0.00^a^	0.00 ± 0.00^a^	0.00 ± 0.00^a^	0.00 ± 0.00^a^	0.00 ± 0.00^a^	0.00 ± 0.00^a^
5 days	0.00 ± 0.00^a^	0.00 ± 0.00^a^	0.00 ± 0.00^a^	0.00 ± 0.00^a^	0.00 ± 0.00^a^	0.00 ± 0.00^a^
Male						
1 day	0.00 ± 0.00^a^	1.89 ± 0.33^c^	0.98 ± 0.50^b^	1.06 ± 0.22^bc^	0.70 ± 0.32^b^	0.48 ± 0.41^ab^
2 days	0.00 ± 0.00^a^	0.21 ± 0.19^b^	0.00 ± 0.00^a^	0.02 ± 0.04^a^	0.00 ± 0.00^a^	0.00 ± 0.00^a^
3 days	0.00 ± 0.00^a^	0.16 ± 0.28^^a^^	0.00 ± 0.00^a^	0.00 ± 0.00^a^	0.00 ± 0.00^a^	0.00 ± 0.00^a^
4 days	0.00 ± 0.00^a^	0.00 ± 0.00^a^	0.00 ± 0.00^a^	0.00 ± 0.00^a^	0.00 ± 0.00^a^	0.00 ± 0.00^a^
5 days	0.00 ± 0.00^a^	0.00 ± 0.00^a^	0.00 ± 0.00^a^	0.00 ± 0.00^a^	0.00 ± 0.00^a^	0.00 ± 0.00^a^

**Table 3:** Effect of MPP on cecal microflora in female and male mice. The values are presented as means ± standard deviation (10 mice/group). ^a–c^Data within a row without the same superscripts are different significantly (*P* < 0.05).

Groups	Normal	Negative	POS	MPP-L	MPP-M	MPP-H
Female						
*Bifidobacterium* (lgCFU/g)	8.42 ± 0.17^b^	7.68 ± 0.04^a^	8.76 ± 0.44^bc^	8.53 ± 0.39^b^	9.10 ± 0.08^c^	7.80 ± 0.29^a^
*Lactobacilli* (lgCFU/g)	8.90 ± 0.16^b^	7.86 ± 0.47^a^	8.62 ± 0.31^b^	8.63 ± 0.23^b^	8.49 ± 0.16^b^	7.96 ± 0.20^a^
*Enterococcus* (lgCFU/g)	6.88 ± 0.45^a^	8.45 ± 0.74^c^	8.08 ± 0.35^c^	8.37 ± 0.10^c^	8.01 ± 0.03^c^	7.65 ± 0.08^b^
*E. coli* (lgCFU/g)	4.92 ± 0.15^a^	6.52 ± 0.40^c^	4.63 ± 0.13^a^	5.23 ± 0.22^ab^	4.98 ± 0.63^ab^	4.67 ± 0.17^a^
*Enterobacterium* (lgCFU/g)	5.56 ± 0.38^a^	7.21 ± 1.08^b^	5.14 ± 0.41^a^	5.38 ± 0.19^a^	5.26 ± 0.28^a^	4.83 ± 0.14^a^
*B*/*E*	1.51	1.07	1.70	1.59	1.73	1.61
Male						
*Bifidobacterium* (lgCFU/g)	8.63 ± 0.17^ab^	7.77 ± 0.05^a^	8.50 ± 0.59^ab^	7.92 ± 0.93^a^	8.87 ± 0.08^b^	8.14 ± 0.35^ab^
*Lactobacilli* (lgCFU/g)	9.11 ± 0.07^b^	8.65 ± 0.15^ab^	8.65 ± 0.19^^ab^^	8.41 ± 0.23^ab^	8.56 ± 0.30^ab^	7.97 ± 0.76^a^
*Enterococcus* (lgCFU/g)	6.54 ± 0.08^a^	7.70 ± 0.25^a^	7.13 ± 1.42^a^	7.38 ± 0.24^a^	7.78 ± 0.22^a^	7.70 ± 0.18^a^
*E. coli* (lgCFU/g)	5.21 ± 0.39^ab^	6.02 ± 0.02^b^	4.80 ± 0.10^a^	5.68 ± 0.61^b^	5.06 ± 0.14^a^	4.81 ± 0.25^a^
*Enterobacterium* (lgCFU/g)	5.86 ± 1.37^a^	6.87 ± 0.11^c^	5.01 ± 0.35^a^	6.03 ± 0.80^a^	5.58 ± 1.31^a^	4.96 ± 0.48^a^
*B*/*E*	1.47	1.13	1.70	1.31	1.60	1.64
